# Seasonal variation of the dominant allergenic fungal aerosols – One year study from southern Indian region

**DOI:** 10.1038/s41598-017-11727-7

**Published:** 2017-09-11

**Authors:** Hema Priyamvada, Raj Kamal Singh, M. Akila, R. Ravikrishna, Rama Shanker Verma, Sachin S. Gunthe

**Affiliations:** 1 EWRE Division, Department of Civil Engineering, Indian Institute of Technology Madras, Chennai, 600036 India; 2 Department of Chemical Engineering, Indian Institute of Technology Madras, Chennai, 600036 India; 3 Department of Biotechnology, Indian Institute of Technology Madras, Chennai, 600036 India

## Abstract

Quantitative estimations of fungal aerosols are important to understand their role in causing respiratory diseases to humans especially in the developing and highly populated countries. In this study we sampled and quantified the three most dominantly found allergenic airborne fungi, *Aspergillus fumigatus*, *Cladosporium cladosporioides*, and *Alternaria alternata* from ambient PM_10_ samples using the quantitative PCR (qPCR) technique in a southern tropical Indian region, for one full year. Highest concentrations of *A*. *fumigatus* and *C*. *cladosporioides* were observed during monsoon whereas *A*. *alternata* displayed an elevated concentration in winter. The meteorological parameters such as temperature, relative humidity, wind speed, and precipitation exhibited a substantial influence on the atmospheric concentrations of allergenic fungal aerosols. The morphological features of various allergenic fungal spores present in the PM_10_ were investigated and the spores were found to possess distinct structural features. In a maiden attempt over this region we correlate the ambient fungal concentrations with the epidemiological allergy occurrence to obtain firsthand and preliminary information about the causative fungal allergen to the inhabitants exposed to bioaerosols. Our findings may serve as an important reference to atmospheric scientists, aero-biologists, doctors, and general public.

## Introduction

Fungi are one of the most important microorganisms that constitute a major fraction (by mass and number) of the atmospheric aerosol particles. They are ubiquitously found in both the outdoor and indoor environments and, many fungi are known to exert type I hypersensitivity reactions including allergic rhinitis and allergic asthma in healthy and sensitive human beings^1, 2^. Respiratory diseases in humans associated with the aerosol particle exposure are increasing rapidly with 300 million asthmatics worldwide and interestingly 1/10^th^ of the world asthmatics belong to Indian sub-continent^3, 4^. According to the WHO (World Health Organization), approximately 15 – 20 million people in India have asthma attributable to the outdoor air exposure^5–7^. The prevalence of fungi associated respiratory allergies has been estimated to be 20 – 30% among the atopic individuals and up to 6% for the general population^5^. Some of the most common respiratory diseases caused by fungi to humans are invasive pulmonary aspergillosis, chronic obstructive pulmonary disease, allergic fungal sinusitis, and fungal allergic ear infections^8–11^. Inhalation of fungal aerosols can affect the humans in three major ways, (i) by causing infectious diseases, (ii) exerting and exacerbating allergies, and (iii) causing mycotoxin induced severe toxic reactions eventually at times leading to death in immuno-compromised patients^1, 9, 12^. Mycotoxins produced by fungi are toxic to both humans and animals mainly through the inhalation and ingestion. They have been reported to cause medical complications to livers, kidneys, gastrointestinal tract, heart, central nervous system, and the immune system. Mycotoxins released by several fungi have been reported to be carcinogenic^13^. The most common fungal allergens are *Aspergillus*, *Cladosporium*, *Alternaria*, *Penicillium*, and yeasts^14^. While the threshold levels of sensitization and asthma are available for dust mite allergens, such values are still unavailable for fungi, especially the fungal aerosols, despite their ubiquitous presence in the atmosphere^8, 11, 12, 15^.

The unavailability of the data for the allergenic fungal aerosols is mainly due to the lack of application of advanced techniques to quantify the presence of outdoor fungi as a part of atmospheric bioaerosols. Till date, majority of the studies that have reported the diversity of outdoor fungi were largely based on the traditional culture techniques^16–20^. Few studies, however have reported the diversity of fungi in the temperate regions of the world using the molecular biological techniques^21–26^. The quantification of atmospheric fungi based on culture techniques are often reported to be biased as many fungi are incapable of growing in the selective growth media^11, 22, 25, 27^. Additionally, culture-dependent methods cannot detect non-viable fungi, which are still of clinical importance as they retain their allergenic biomolecules such as ergosterol and proteins^11, 12, 28^. Furthermore, the identification and diversity estimations based on the cultivation techniques are considered to be an underestimation of the actual outdoor fungal concentrations^22, 27^. In contrast to the existing culture-based quantification techniques, emerging advanced molecular-based methods including quantitative PCR are not dependent on the fungal viability and can lead to better estimations by 2 – 3 orders of magnitude^15, 25, 26, 29^. As the conventional cultivation techniques possesses several disadvantages, it is now pertinent to adopt advanced techniques for the rapid and reliable detection, and quantification of allergenic fungi in the atmosphere to effectively quantify their role in causing allergies.

In this study we investigate the three most dominantly reported allergenic and plant pathogenic fungi, *Aspergillus fumigatus* (*AF*), *Cladosporium cladosporioides* (*CC*) and *Alternaria alternata* (*AA*) in the ambient PM_10_ using the advanced molecular based real time PCR (RT-PCR) technique. The objectives of this study are, (i) to investigate the prevalence and report the concentrations of the three different allergenic fungi in the atmospheric aerosols for one full year covering three seasonal cycles of summer (Feb – May), monsoon (June – Sep), and winter (Oct – Jan), (ii) to statistically determine the difference in the allergenic fungal abundance and to analyze the effect of meteorological parameters on their ambient concentrations, and (iii) morphological characterization of allergenic fungal spores using scanning electron microscope (SEM) imaging analysis. Further, for the first time we have correlated the ambient allergenic fungal concentrations with the prevalence of reported allergy cases over the study region.

## Results and Discussion

The RT-PCR analysis of the pooled DNA extracts of all the twelve months analyzed (July 2014 – June 2015) involving 53 air filter samples, revealed the presence of three allergenic fungi, *A*. *fumigatus*, *C*. *cladosporioides*, and *A*. *alternata*, throughout year. The SYBR green real-time PCR analyses gave strong positive fluorescence signals between 13 to 25 cycles and faint signals after 30^th^ cycle. With the Ct value obtained from the RT-PCR runs, the allergenic fungi concentrations for each month were calculated and represented as DNA copies m^−3^ of air. It should however be noted that from the gene copy numbers m^−3^, the number of atmospheric fungal spores cannot be obtained as the number of cells per spore vary from species to species^15^ and hence it becomes difficult to compare the concentrations among different species^15, 30^. To overcome this problem in future, we suggest performing the DNA calibration curves with the spores obtained from the specific species. Nevertheless, this study provides firsthand information about the atmospheric concentrations of the most dominantly found allergenic fungi over the Indian region. Among the three allergenic fungi quantified, *C*. *cladosporioides* was the most abundantly found fungi with its concentrations being one order of magnitude higher than *A*. *fumigatus* and two orders of magnitude higher than *A*. *alternata*. Several other studies from various regions across the globe that have investigated the diversity of air mycoflora have similarly reported *Cladosporium* to be the most frequently occurring and dominant species in the ambient air^22, 31, 32^. In our study, the difference in the distribution of the three allergenic fungi was statistically verified by performing the paired t-test and one-way ANOVA followed by Games-Howell post hoc test. From the paired t-test, it was found that *A*. *fumigatus* was having a greater distribution than *A*.*alternata* (p < 0.05) for all the three seasons. Further, from the paired t-test performed for *A*. *fumigatus* Vs. *C*. *cladosporioides* and *C*. *cladosporioides* Vs. *A*. *alternata*, *C*. *cladosporioides* was found to possess a greater distribution (p < 0.05) in both the cases. With the p value (p < 0.05) obtained from the one-way ANOVA and Games-Howell post hoc test it was seen that the concentration distribution of all the three allergenic fungi varied from each other, all through the year. The p-values obtained from the Games-Howell post hoc test could be found in the supplementary material (see supplementary Table S1).

In our study, we found all of the three allergenic fungi to exhibit a significant variation in the concentrations during all the three seasons and the details are discussed below.

### Aspergillus fumigatus

*A*. *fumigatus* is one of the most ubiquitously found airborne fungi; however they have been widely reported to be present in the soil and decaying organic matter^33^. *A*. *fumigatus* has been reported to sporulate abundantly and every single conidium of the fungi releases thousands of spores into the atmosphere^34^. It has also been reported that humans inhale at least several hundred *A*. *fumigatus* spores every day^35^ and the continuous inhalation of these spores by immuno-compromised individuals results in the manifestation of invasive lung aspergillosis^36^. Additionally, it has also been reported that *A*. *fumigatus* is the most common agent in causing ~90% of the human respiratory infections^34, 35^.

**Figure 1:**
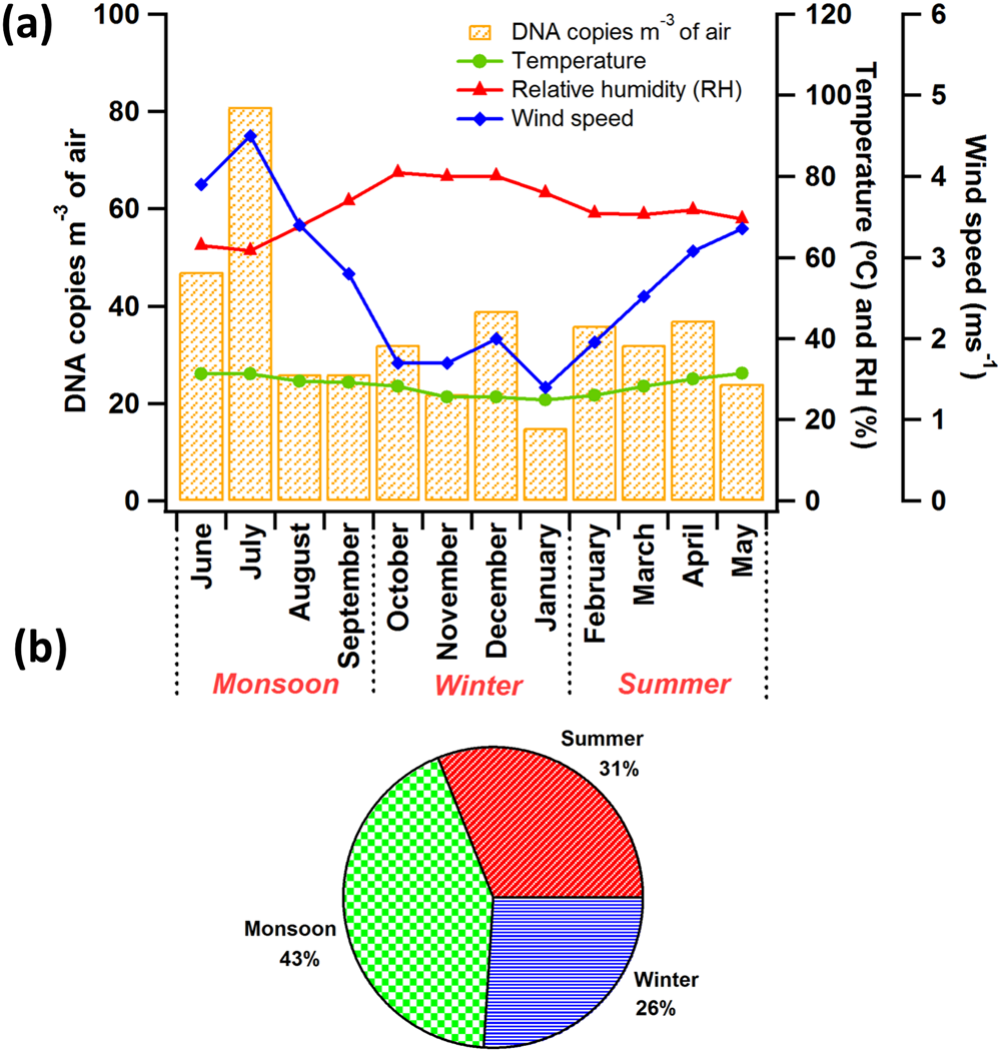
Monthly (**a**) and seasonal (**b**) variations in the concentrations of *A*. *fumigatus* quantified from the qPCR analysis. Concentrations have been represented in terms of DNA copies m^−3^ of air. Concentrations have been represented along with various meteorological parameters such as temperature (°C), humidity (%), and wind speed (ms^−1^). *A*. *fumigatus* was found to be highest during the months of monsoon (43%; June – Sep).

In our study, we have observed the presence of *A*. *fumigatus* in all the air filter samples analyzed. The concentration variation of *A*. *fumigatus* for the entire year (July 2014 – June 2015) is depicted in Fig. 1a. The concentrations (DNA copies m^−3^ of air) were observed to be fairly constant throughout the year (34.8 ± 16.8) with January and July exhibiting the lowest (15 DNA copies m^−3^ of air) and highest (81 DNA copies m^−3^ of air) concentrations, respectively (Table 1). The first half of the monsoon (45.1 ± 25.6) exhibited high concentration of DNA copies, which subsequently decreased during the second half of monsoon and by the beginning of winter. The corresponding temperature (°C) during the same period remained fairly constant with slight decrease from monsoon (30.4 ± 1.1) to winter (26.1 ± 1.4). The relative humidity (%) on the other hand exhibited considerable increase from monsoon to winter season (monsoon: (66.7 ± 5.5) and winter: (79.3 ± 2.2)). High concentrations of *A*. *fumigatus* observed during the months of July and June could be attributed mainly to low humidity levels (60%), high temperature (>30 °C), and increased wind speed (4 ms^−1^) that prevailed during these months. *A*. *fumigatus* belongs to the ‘dry air spora’ category^37, 38^, which is, however, known to release the spores by both ‘active’ and ‘passive’ release mechanism^39^. The spores of Ascomycetes are known to ‘actively’ discharge their spores when their ascus (the turgid sac like cells that contain the spores) gets pressurized osmotically, which then leads to a violent liberation of spores^39–41^. The variation in the turgor pressure of the ascus is controlled by its water content. The ascus is said to rupture when the water content inside it increases by water absorption from the atmospheric moisture (humidity) and when the water content decreases due to the evaporation from the ascus (at high atmospheric temperature)^41^. On the other hand, when the spores get dislodged from the conidiophore due to the external disturbances caused by air currents or by insect landing on the fungi, then such a release mechanism is termed as a ‘passive’ release^39, 42^. Thus, higher temperature (>30 °C) and lower relative humidity (<70%) are primarily responsible for the active release^34, 43^ whereas higher wind speed plays an important role in passive release of the spores^34, 44^. Our findings reported in this study are also in line with these observations where elevated concentrations of *A*. *fumigatus* were observed during the dry period and elevated temperatures (Fig. 1a).

**Table 1:** Concentrations of three allergenic fungi represented along with meteorological parameters.

Parameters		Monsoon	Winter	Summer
Temperature (°C)		(30.4 ± 1.1)	(26.1 ± 1.4)	(29.0 ± 2.4)
Relative humidity (% RH)		(66.7 ± 5.5)	(79.3 ± 2.2)	(70.8 ± 0.9)
Wind (km/h)		(13 ± 2.6)	(6 ± 0.81)	(9.8 ± 2.2)
Rainfall (mm)		(43.5 ± 30.3)	(65 ± 57.4)	(34.7 ± 52.3)
Concentration (DNA copies/m^3^ of air)	*AF*	(45.1 ± 25.6)	(27.1 ± 10.4)	(32.3 ± 6.2)
	*CC*	(869.1 ± 677.7)	(411 ± 383.7)	(491.6 ± 422.4)
	*AA*	(2.5 ± 1.3)	(3 ± 1.4)	(1.3 ± 0.5)

Seasonal average values are represented along with their standard deviation.

Furthermore, ‘dry spore discharging’ fungi require relatively higher temperature (>30 °C) for their growth, sustenance, and spore release into the atmosphere^45^. The low temperature (25 °C) that existed during January could be unfavorable for the proliferation and subsequent spore release of *A*. *fumigatus* ^46^. Further, the role of precipitation was not seen to significantly influence the spore release and thus the concentrations of *A*. *fumigatus* (see supplementary Fig. S1(a)). The seasonal variation thus indicated *A*. *fumigatus* to be highest during monsoon (south-west) and was observed to follow the pattern of, monsoon (43%) > summer (31%) > winter (26%) (Fig. 1b). Various studies that have reported the fungal air spora diversity from different locations of the world have also shown the elevated presence of *Aspergillus* during the dry periods and our findings are also in accordance with this trend^47–50^.

### Cladosporium cladosporioides

**Figure 2:**
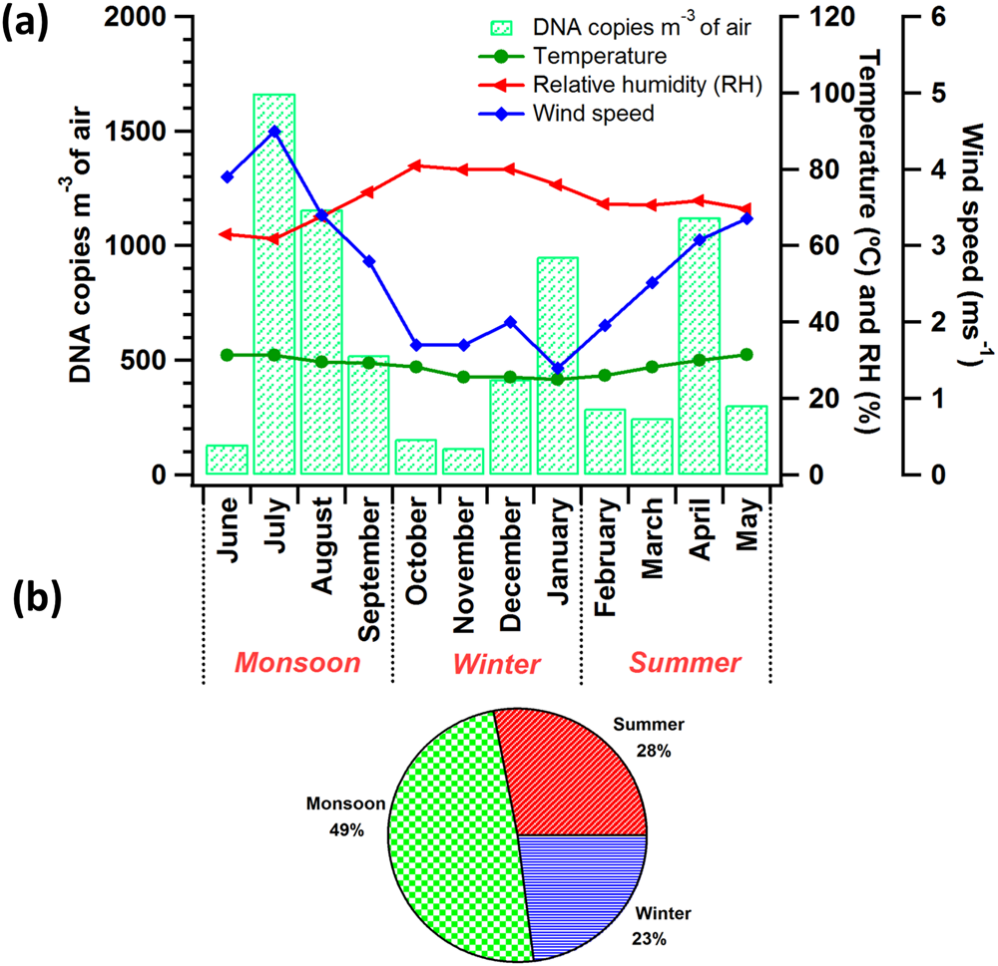
Monthly (**a**) and seasonal (**b**) variations in the concentrations of *C*. *cladosporioides* quantified from the qPCR analysis. Concentrations have been represented in terms of DNA copies m^−3^ of air. Concentrations have been represented along with various meteorological parameters such as temperature (°C), humidity (%), and wind speed (ms^−1^). *C*. *cladosporioides* was found to be highest during the months of monsoon (49%; June – Sep).

*Cladosporium*, a widely known asthmatic fungus, has been reported as one of the most widespread and abundantly found airborne fungus across the globe^9, 11, 14, 22^, with the records showing *C*. *herbarium*, *C*. *macrocarpum* and *C*. *cladosporioides* as the most commonly encountered species in the outdoor ambient environment^1^. The culture-dependent studies performed to estimate the airborne concentrations of *Cladosporium* in the various regions of Europe and North America have reported a year-long presence of *Cladosporium* in the ambient atmosphere^51–54^. Among the three allergenic and plant pathogenic fungi investigated, *C*. *cladosporioides* was found to be the most abundantly present fungi with concentrations exceeding 1500 DNA copies m^−3^ of air (Fig. 2; Table 1). As shown in Fig. 2a, the ambient concentration of *C*. *cladosporioides* similar to *A*. *fumigatus* was highest during July (1664 DNA copies m^−3^ of air; Fig. 2), and was lowest during November (117 DNA copies m^−3^ of air; Fig. 2). *C*. *cladosporioides* also belong to the ‘dry air spora’^38, 45, 55^ and hence their concentrations in the ambient atmosphere were seen to be high during the dry months (July to September). The meteorological conditions such as relative humidity and wind speed that existed during those dry months (low relative humidity −60% and high wind speed −4 ms^−1^) were favorable for the spore release. Further, the concentrations were found to be the lowest during November, the month with highest relative humidity (80%) and lowest wind speed (1.6 ms^−1^). In addition, few pronounced variations in the ambient concentrations of *C*. *cladosporioides* were observed in the months of April, and August. It was seen that during these two months an elevated concentration of *C*. *cladosporioides* was observed and this increase in concentration can be attributed to the release of the spores triggered by the mechanical action resulting from the precipitation (see supplementary Fig. S1(b)). This phenomenon, wherein the rainfall causing an increase of the fungal spore concentrations in the atmosphere is called as the ‘splash induced spore release of fungi’ and this role of rainfall in elevating the fungal concentrations in the atmosphere has been reported from various other studies^37, 44, 56–58^. Consequently, in our study we could see the predominant influence of relative humidity, temperature, and wind speed on the concentrations of *C*. *cladosporioides* in air, throughout the year. However, during April and August the spore release of *C*. *cladosporioides* could have been governed by rainfall along with decreased relative humidity, increased temperature, and increased wind speed. Similarly abundance of *Cladosporium* predominantly during dry periods and rainy periods have also been reported in other regions of the world^37, 59^.

**Table 2:** Primer details of the three allergenic fungi.

Fungal species	Primer name	Sequence 5′–3′
*Alternaria alternata*	AaltrF1	GGCGGGCTGGAACCTC
	AltrR1-1	GCAATTACAAAAGGTTTATGTTTGTCGTA
*Aspergillus fumigatus*	AfumiF1	GCCCGCCGTTTCGAC
	AfumiR1	CCGTTGTTGAAAGTTTTAACTGATTAC
*Cladosporium cladosporioides*	Cclad2F1	TACAAGTGACCCCGGCTACG
	CcladR1	CCCCGGAGGCAACAGAG

The implication of variations in ambient relative humidity on concentration of *Cladosporium* is as such complicated to quantify. For example Kurkela (1997)^60^ has reported high concentration of *Cladosporium* when the relative humidity was in the range of 40 – 70% with wind velocities in the range of 3 – 5 ms^−1^. The concentration was found to be significantly higher when the relative humidity ranged between 60 to 65%. His study also reported the impact of precipitation on *Cladosporium* concentration and described to have the impact on following two ways: firstly, rainfall provides ample moisture required for the mycelial growth and spore production during the rainy periods. Secondly, as mentioned above, rainfall serves as a force inducing the release of the spores from the aerial hypha. Interestingly, a combination of optimal meteorological parameters such as relative humidity (optimal RH – 67% for spore release was observed in monsoon), wind speed (optimal wind speed – 4 ms^−1^ for spore release was observed in monsoon), and rainfall could have caused the higher concentrations over this study region during those specific two months mentioned above (refer the methodology for the details about the study location). Seasonal variation in the occurrence of *C*. *cladosporioides* was as follows (49%) > summer (28%) > winter (23%) and is shown in Fig. 2b.

### Alternaria alternata

**Figure 3:**
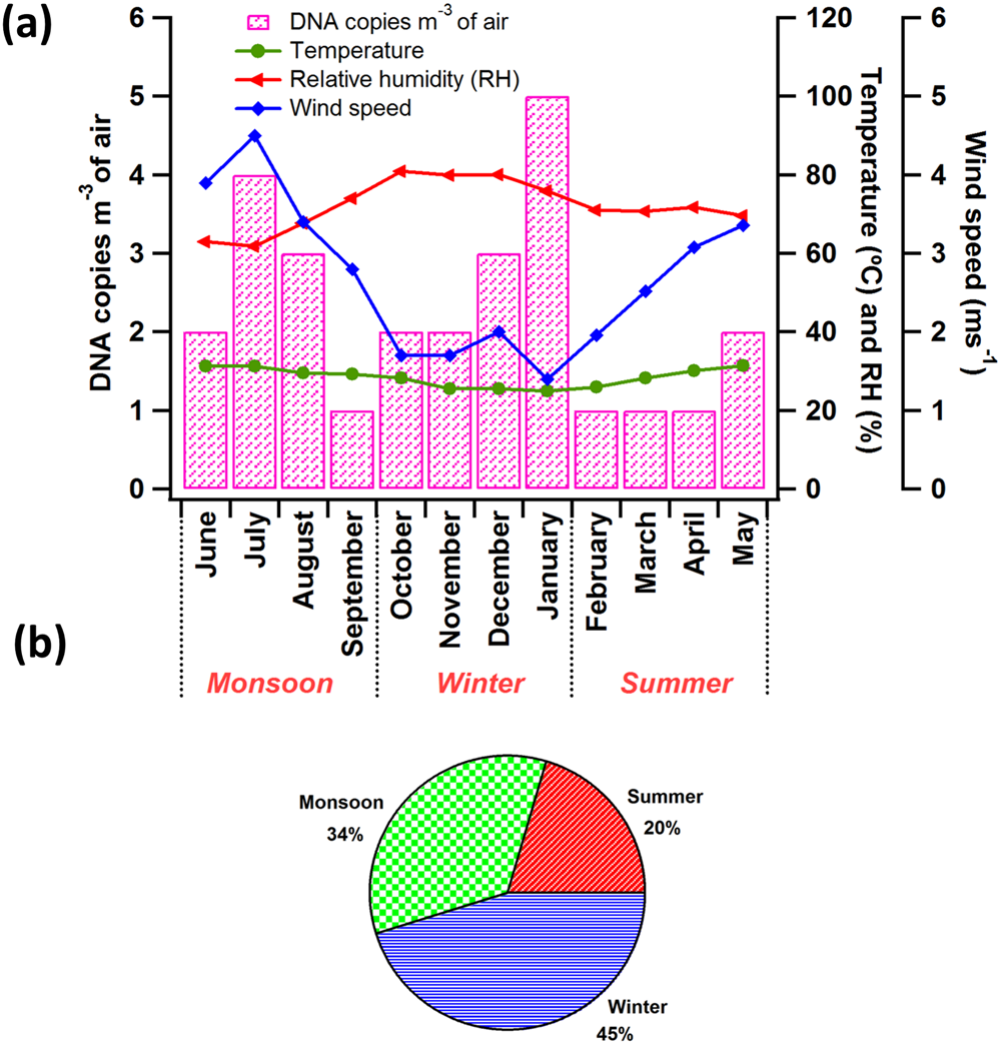
Monthly (**a**) and seasonal (**b**) variations in the concentrations of *A*. *alternata* quantified from the qPCR analysis. Concentrations have been represented in terms of DNA copies m^−3^ of air. Concentrations have been represented along with various meteorological parameters such as temperature (°C), humidity (%), and wind speed (ms^−1^). *A*. *alternata* was found to be highest during the months of winter (45%; Oct – Jan).

*A*. *alternata* occurs primarily on plants, soil, decaying organic matter, and its most favorable habitat has been reported as the forest plants^61^. *A*. *alternata* is known to affect both plants and humans where it causes ‘leaf spot syndrome’ in plants^62, 63^ and upper respiratory tract infections in humans^8, 64^. Among the various *Alternaria* species, *A*. *alternata* is considered as an important airborne allergen with their conidial spores and mycelial fragments being responsible for the allergic reactions in patients with rhinitis and asthma^1, 14, 65^. It’s existence has been reported in both the wet and dry periods of the temperate regions^37, 63, 64, 66^. The concentrations of *A*. *alternata* were found to be the lowest in the study region over the entire sampling period in comparison to the other two allergenic fungi investigated. *A*. *alternata* reached its highest concentrations in winter months, with maximum levels in January (5 copies m^−3^ of air) (Fig. 3a). Since the concentrations obtained were very low, the significant influence of meteorological parameters on the concentrations cannot be robustly elucidated. However, the possible role of certain meteorological parameters on influencing the ambient concentrations of *A*. *alternata* has been discussed in the following lines and the relations are speculative in nature. The average spore concentration during monsoon was (2.5 ± 1.3) DNA copies m^−3^ of air, which increased by 20% during the winter season. Highest concentrations of *A*. *alternata* during winter coinciding with highest average humidity (76%), rainfall (average value of 65mm), and wind speed (~1 ms^−1^) amongst all three seasons (Table 1). Additionally, it has been reported that the optimal temperature for the growth and spore release of *Alternaria* is usually in the range of 20 – 28 °C^67^ as observed in the present study (Table 1). Thus, the high spore concentrations during winter could be attributed to the active release of spores due the high relative humidity during winter. Further, precipitation was not seen to significantly influence the concentrations of *A*. *alternata* (see supplementary Fig. S1(c)). Though not many studies are available about the ambient concentrations of *A*. *alternata*, a study performed by Gofroń, G. A., 2011, has reported *Alternaria* to be highest during the dry periods over the regions of Poland^68^. However, contrasting observations have been reported where the elevated concentrations of *Alternaria* was observed during rainy periods^69, 70^. The seasonal variation in the ambient occurrence of *A*. *alternata* depicted the following trend, winter (45%) > monsoon (34%) > summer (21%) (Fig. 3b).

#### Relationship between ambient concentrations of allergenic fungi and meteorological parameters

**Figure 4:**
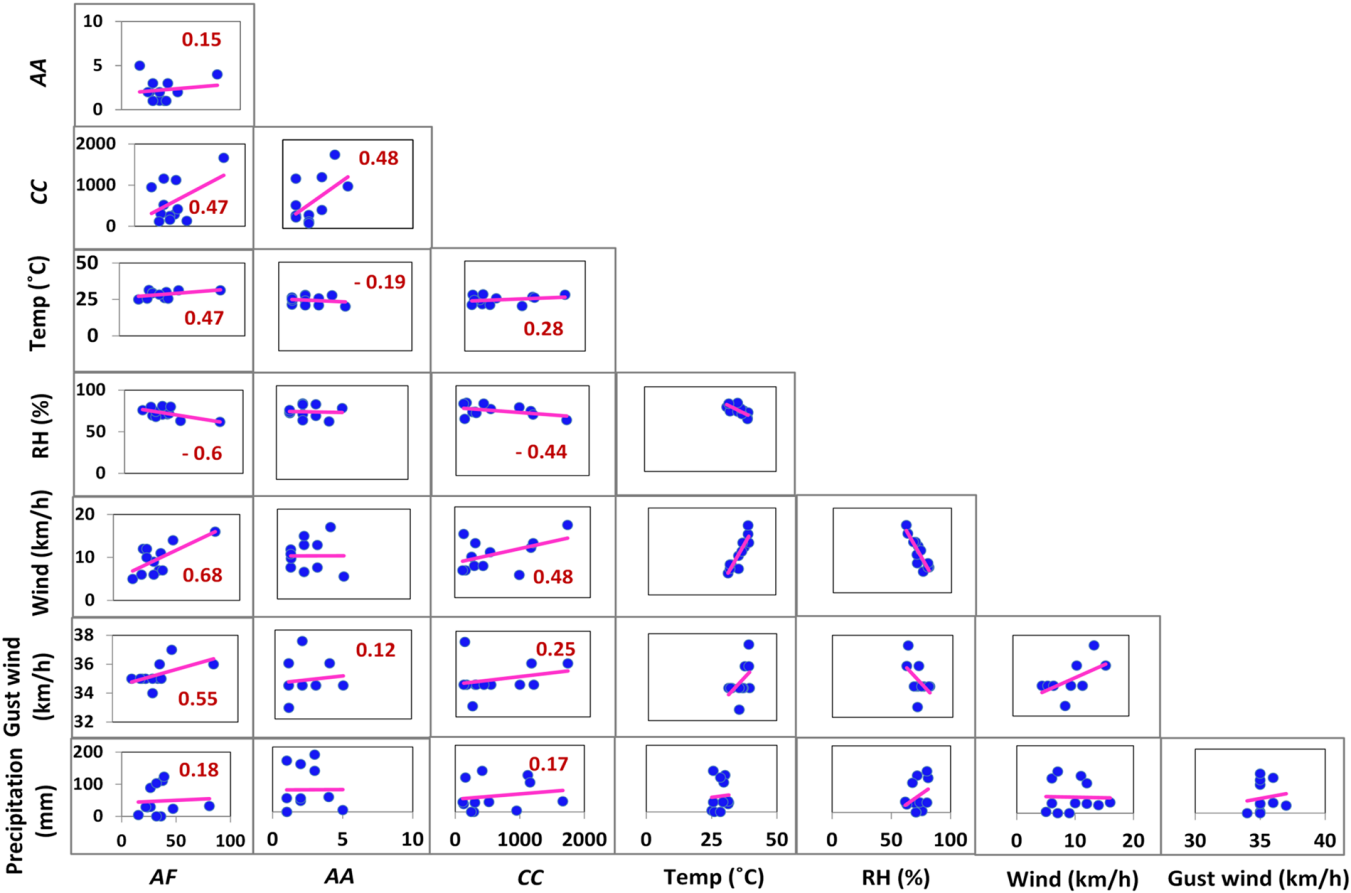
Effect of meteorological parameters on ambient fungal concentrations. Scatter plot matrix depicting the correlation of the concentrations of three allergenic fungi *A*. *fumigatus* (*AF*; DNA copies m^−3^ of air), *A*. *Alternaria* (*AA*; DNA copies m^−3^ of air), and *C*. *cladosporioides* (*CC*; DNA copies m^−3^ of air) with the meteorological parameters – temperature (°C), relative humidity (%), wind speed (depicted as wind in the matrix; kmh^−1^), and wind gust (kmh^−1^).

The assessment of the effect of meteorological parameters on the allergenic fungi is very important^71–73^ as several meteorological parameters largely influence the release, dispersal, and sustenance of the fungal spores in the atmosphere after their release from the parental bodies. A scatter plot matrix (Fig. 4) generated by performing the correlation matrix analysis based on linear regression depicts the influence of temperature (°C), relative humidity (%), wind speed (ms^−1^), wind gust (ms^−1^), and precipitation (mm) on the allergenic fungi concentrations (DNA copies m^−3^ of air). As shown in Fig. 4, it is evident that *A*. *fumigatus* showed a good positive linear relation (r = 0.47) with *C*. *cladosporioides* and *C*. *cladosporioides* showed a good positive linear relation (r = 0.48) with *A*. *alternata* indicating the co-occurrence and viability of these two fungal pairs in the environmental conditions existing in the study region. However, the correlation value (r = 0.15) obtained for *A*. *fumigatus* and *A*. *alternata* was insignificant indicating a different trend in the occurrence of *A*. *fumigatus* and *A*. *alternata* fungal air spora in the ambient atmosphere. Interestingly, relative humidity exhibited a notable negative correlation with *A*. *fumigatus* (r = −0.60) and *C*. *cladosporioides* (r = −0.44), further confirming the trivial role of relative humidity on the occurrence of these dry air spora. Further, as shown previously temperature indeed exhibited a good positive correlation (r = 0.47) and a moderately positive correlation (r = 0.28) with the dry air spora, *A*. *fumigatus* and *C*. *cladosporioides*, respectively. Among, all the meteorological variables that were analyzed, wind speed were seen to prominently influence *A*. *fumigatus* (r = 0.68) and *C*. *cladosporioides* (r = 0.48), with a notable positive correlation. The wind gust also exhibited a positive correlation (r = 0.55 for *A*. *fumigatus* and r = 0.25 for *C*. *cladosporioides*) with the two dry air spora, however the correlation values obtained were relatively lower compared to the wind speed. This reveals that high wind speed >8 ms^−1^ may inhibit the fungal spore release from conidia^40, 42^. *A*. *alternata* was seen to exhibit a negative correlation with temperature whereas, a positive correlation with relative humidity, wind, and wind gust. However, all the correlation values obtained for *A*. *alternata* were insignificant to draw any conclusions towards identifying the role of meteorological parameters on concentration of *A*. *alternata*. Furthermore, all of the three allergenic fungi exhibited an insignificant correlation (r < 0.15) with the precipitation. Thus from the inter-relationships obtained it could be seen that temperature, wind speed, and wind gust have a notable influence on the occurrence of *A*. *fumigatus* and *C*. *cladosporioides*, whereas the impact of relative humidity was observed mixed in nature for *A*. *alternata*.

#### Morphological characterization of allergenic fungal spores

The morphological characterization of fungal spores is essential to understand and derive their aerobiological pathway in the atmosphere^74, 75^. Also their structural details help in modeling the fungal spore exposure to human respiratory system. Using both the culture-dependent and culture-independent molecular techniques various types of allergenic and plant pathogenic fungal spores present in the atmospheric PM_10_ were identified up to the species level.

**Figure 5:**
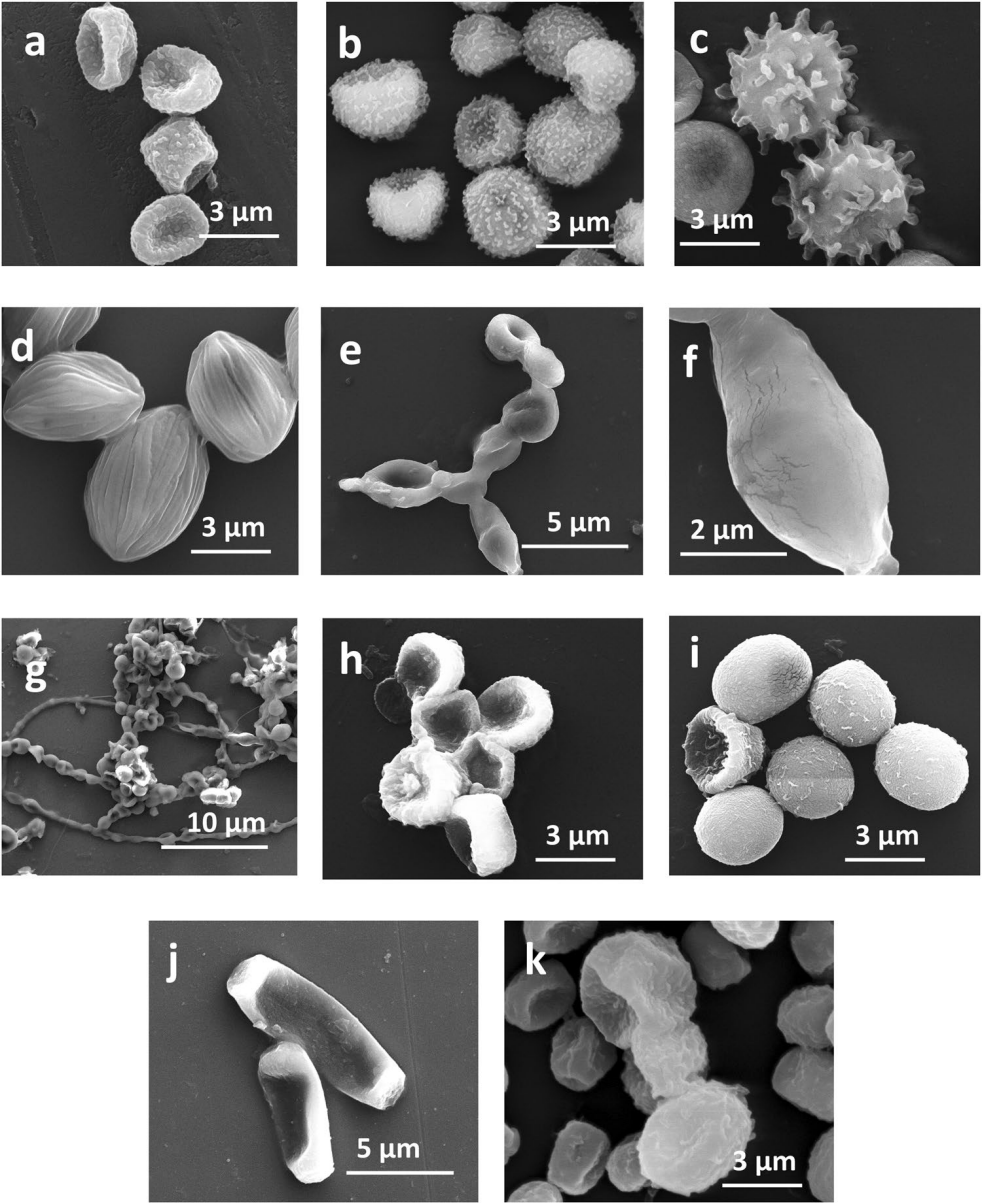
SEM images of allergenic and plant pathogenic fungal spores. DNA analysis of the spores revealed their species type, (**a**) *A*. *fumigatus*, (**b**) *A*. *flavus*, (**c**) *A*. *niger*, (**d**) *A*. *rhizopus*, (**e**) young spores of *Cladosporium sp*. in a chain form, (**f**) mature single spore of *Cladopsorium sp*., (**g**) *Alternaria sp*., (**h**) *Rhizopus sp*., (**i**) *Chaetomium sp*., (**j**) *Neurospora crassa*., and (**k**) *Epicoccum sp*. Scale is different for every image and is given at the bottom.

From the SEM imaging, we found that the size range of the allergenic fungal spores varied between 3 and 8 µm (equivalent aerodynamic diameter 2 – 5 µm)^76^. They mostly appeared to be ovoid, globular, sub-globular, and elongated and were having either a smooth surface or an ornamented surface. Additionally, many of the spores were found to possess the attachment scar at their anterior and/or posterior ends. In addition to the three dominant allergenic fungi (*A*. *fumigatus*, *C*. *cladosporioides* and *A*. *alternata*) quantified in this study, we have identified and studied the morphological features of the following allergenic and plant pathogenic fungi, *Aspergillus niger*, *Aspergillus flavus*, *Aspergillus rhizopus*, *Alternaria sp*., *Rhizopus sp*., *Chaetomium sp*., *Eurotium sp*., and *Neurospora crassa* (Fig. 5). Apart from elaborately studying the morphological features of the fungal spores, the structural features of the conidia before and after their spore release was also morphologically analyzed (see supplementary Fig. S2).

#### Correlations between the epidemiological allergy incidence and the ambient allergenic fungi concentrations

Owing to the ubiquitous presence of fungi in the atmosphere, their role as an allergen source is almost inevitable. In cases where the clinical data are supplemented with studies about the patient’s environment, revealing the fungal allergen concentrations in the air, there is more certainty about type of fungi actually causing the allergy to exposed inhabitants of a region under investigation. With this motive a correlation analysis was performed between the incidence of allergy in the residents of this study region and the ambient allergenic fungi concentrations that were elucidated. We believe that this analysis would provide preliminary information about the probable ambient allergenic fungal sources that could potentially incite allergies to the residents when there is an increase in their ambient concentrations. However, the information obtained from this study has to be further complemented with clinical experimental analysis such as ‘sera-based immunoassays’, to draw robust conclusion on finding the actual causative fungal allergen. It is important to note that the conclusions obtained from this analysis is region specific and should not be extrapolated or compared with the studies giving the information based on physiological relation between presence of allergenic fungi and actual allergies to the inhabitants in a given region. Further, in order to draw the additional informative conclusions from this type of study, we suggest the investigations involving long term cohort studies combined with the quantification of air mycoflora.

**Figure 6:**
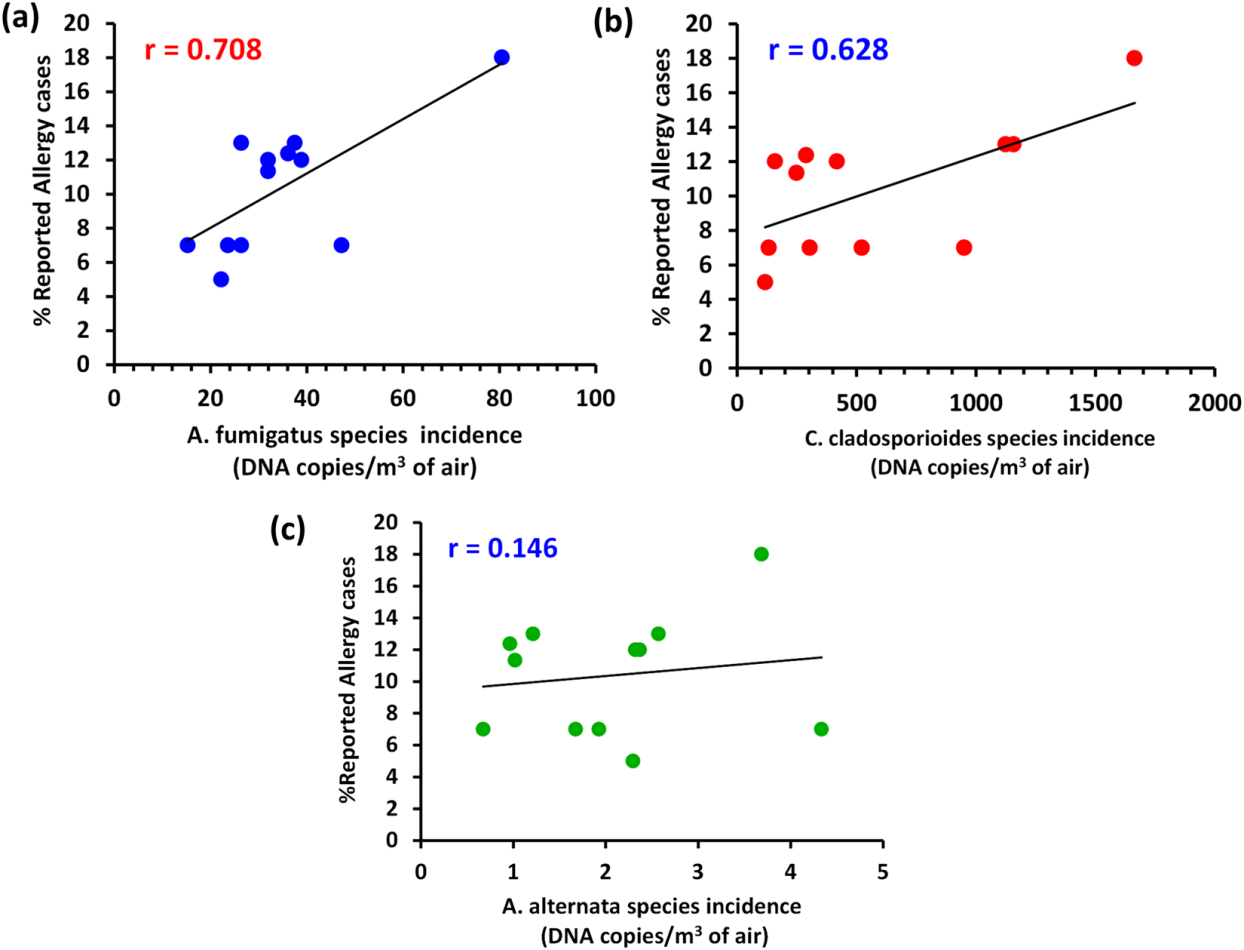
Correlation of allergy incidence with the three ambient allergenic fungi, *A*. *fumigatus* (**a**), *C*. *cladosporioides* (**b**) and *A*. *alternata* (**c**). *A*. *fumigatus* and *C*. *cladosporioides* displayed a good positive correlation with the allergy cases reported over the study region, suggesting their possible role in causing allergies to the inhabitants of the study region. *A*. *alternata* showed insignificant relationship due to the lowest concentrations observed.

The correlation analysis was performed between the reported allergy cases (%) and the allergenic fungal concentrations (DNA copies m^−3^ of air) that were quantified. In general, the reported allergy cases were seen to be highest during monsoon and summer (see supplementary Fig. S3). Incidentally, the ambient concentrations of *A*. *fumigatus* and *C*. *cladosporioides* were also found to be high during the months of summer and monsoon. From Fig. S3 (see supplementary Fig. S3), a qualitative relationship that existed significantly between allergenic fungal concentrations and the reported allergy cases for all the three seasons can be seen. Among all the three allergenic fungi, the allergy cases were seen to follow suit with the concentration variations of *A*. *fumigatus*. Further, from the correlation coefficient (r = 0.708) estimated, (Fig. 6a) a positive and a fairly strong (estimated r > 0.5) relationship between the ambient *A*. *fumigatus* concentrations and the reported allergy cases was seen to exist over this study region. The correlation between *C*. *cladosporioides* and the reported allergy cases yielded a correlation coefficient value of r = 0.63 (Fig. 6b), which further indicated a fairly strong relationship between the fungal incidence and the allergy cases. However, the r value obtained for *A*. *alternata* was very less (r = 0.15) (Fig. 6c) and its influence on the reported allergy cases was not significant compared to the other two allergenic fungi as evident from their lowest concentration. Thus from this preliminary test it is concluded that fungi imperfecti, *A*. *fumigatus* and *C*. *cladosporioides* might have played a role in causing seasonal allergies to the individuals residing in the study region.

## Conclusions

By analyzing the airborne fungal concentrations for a period of one year, we found a significant seasonal variation in the occurrence of the three allergenic fungi, which was further confirmed statistically by performing paired t-test and one-way ANOVA followed by Games-Howell post hoc test. Consistent with the previous studies, *C*. *cladosporioides* was seen to be the most abundantly present allergenic fungi over the study region. *A*. *fumigatus* and *C*. *cladosporioides* were found to have the highest concentrations during monsoon and summer, whereas *A*. *alternata* was seen to have high concentrations during winter. The meteorological parameters like temperature, relative humidity, and wind speed were found to significantly influence the ambient spore concentrations of the allergenic fungi. The sporadic rainfall that occurred in the study region was seen to positively influence the ambient concentrations of *C*. *cladosporioides* during the winter season, which was difficult to quantify. SEM analysis of fungal spores grown out of collected PM_10_ samples based on the morphological characteristics revealed the presence of seven different types of allergenic fungal spores. All the spores were found to be in 3 – 8 µm size range and displayed a remarkable difference in their surface ornamental features. Correlation analysis performed between the ambient allergenic fungal concentrations and the reported allergy cases indicated a fairly strong influence of *A*. *fumigatus* and *C*. *cladosporioides* in causing allergies to residents of this study region. To the best of our knowledge this is the first such study from the Indian sub-continent combining the quantification studies with the allergy epidemiological data. Further, seasonal changes in the reported allergy cases were seen similar to seasonal changes in the ambient concentrations of *A*. *fumigatus*, indicating their possible profound role in inciting allergies to the inhabitants of this study region. Due to many different reasons, the aetiological role of fungi in relation to the allergic diseases still remains as an area that is far from being completely understood. More such studies combining the aerobiological aspects with epidemiological allergy studies and immunoassays are needed to better understand and quantify the role of airborne fungi in causing respiratory diseases especially for the tropical regions like India, where the airborne fungi are believed to be highly abundant and diverse.

## Methods

### Study region and sampling site

The allergenic fungi quantification using RT-PCR was carried out in Chennai, India. Briefly, the city of Chennai is characterized by tropical hot and humid climate and experiences three distinct meteorological seasons, namely summer, monsoon, and winter. Unlike the other major geographical part of India, which receives ~80% of the total annual rainfall during southwest monsoon season (Jun – Sep), Chennai receives majority of its rainfall during the northeast monsoon (Nov – Jan) season. The vegetation found in the Chennai region is predominantly the tropical dry evergreen biome^74, 77^. The sampling site, Indian Institute of Technology Madras (IITM,; 12.99°N, 80.23°E, 6 m amsl – above mean sea level), spreads across 687 acres of which 18% is occupied by human establishment. IITM is covered by a dense population of trees spreading over the entire area with vast varieties of flora and fauna comprising of 36% trees, 24% herbs, and the rest by shrubs, climbers, grasses, and palm trees^77^. The campus consists of nearly 432 species of plants and animals together with more than 300 different species of plants alone. Many groups of organisms such as bryophytes, fungi, spiders, insects, and butterflies are likely to extend the list of species on the campus considering the favorable conditions for their growth and survival.

### Aerosol sampling

Aerosol samples were collected at the roof top (12 meters from the ground) of Mechanical Sciences Block (MSB), IITM, at a height of 1 – 1.5 meters from the surface of the roof, an appropriate height making sure that air being sampled is above the canopy. Particulate matter sampler PM_10_ (APM550 from Envirotech, India) was used to collect the particulate matter on the filter paper. The reason for choosing PM_10_ is as follows. Fungi exist mostly as spores in the ambient atmosphere and they generally fall in the size range of 3 to 10 µm. Further, the dominant size range of fungal spores observed on global scale is said to be of 3 µm^32, 75, 76, 78^. All of the three allergenic fungi targeted in this study also have an average spore size of 3 µm and they clearly fall under the PM_10_ size range. Hence, PM_10_ was collected to quantify the fungal aerosols. Aerosol samples were collected on glass fiber filters (Whatman, Type GF/F, and 47 mm diameter) for one year from July 2014 to June 2015 and a total of 53 filter samples had been collected. The sampler was operated at a total flow rate of ~16.67 L/min and the sampling period was fixed to 7 days, corresponding to a sampled air volume of ~170 – 175 m^3^. To exclude any contamination prior sampling, all the glass fiber filters were sterilized by baking at 370 – 400 °C, 10 – 12 hours. Loaded filters were packed in aluminum foil (prebaked at 300 °C), and stored at −80 °C until experimental analysis. A sampling blank was included for all the subsequent analysis.

### DNA extraction

One of the quadrants of the air filter samples was used for the DNA extraction using a commercial soil DNA extraction kit, (Fast DNA Spin Kit for Soil with Lysing Matrix E), MP Biomedicals^28^ following the protocol provided in the kit. From the sampling blanks and extraction blanks, DNA was extracted following the same protocol and the extracts were used for the RT-PCR analysis. DNA from the filters were extracted in 100 µL of the elution buffer and stored at −20 °C until further experimental analysis.

### RT – PCR analysis

The Agilent Mx3000 P qPCR system was used and the MxPro software was used to control the operations in the PCR system. The master mix for the PCR run involved the following: 10 μL Fast Start Universal SYBR Green master (Roche, product no: 04913850001), with 1x final concentration, 1 μL of 10 μM of each forward and reverse primer^15^ per reaction (Table 2; primers were acquired from Eurofins, India), 5 μL DNA template filled up with nuclease free water (Ambion® RT-PCR Grade Water, Thermo Fisher Scientific) to the total volume of 20 μL per reaction. The PCR protocol was as follows: initial denaturation step at 95 °C for 10 min, followed by the 40 cycles with: denaturation at 95 °C for 20 s, annealing at 58 °C for 20 s, and elongation at 72 °C for 30 sec. The melting curve analysis had the temperature gradient from 60 °C to 95 °C.

Absolute quantification technique using the standard graph method was used to quantitate the allergenic fungi in the DNA extract of the PM_10_. The standard graphs were constructed using the DNA constructs for each of the three allergenic fungi which were considered as the positive samples. The DNA constructs were amplified, cloned and the resulting plasmid DNA was used for the standard graph construction. A standard graph was prepared using positive DNA constructs by varying its concentration from 5 × 10^−7^ ng/µL to 5 ng/µL. Thereafter, the DNA copy numbers of the unknown samples (filter DNA extracts from July 2014 to June 2015 i.e. 12 DNA extracts, each representing a month) were determined using the standard graph.

### Statistical analysis

To understand the difference in the seasonal distribution of three allergenic fungi, *A*. *fumigatus*, *C*. *cladosporioides*, and *A*. *alternata* the paired t-test was performed^74^. Paired t-test compares two population means where the observations in one sample can be paired with observations in the other sample. In our case, the comparisons were made for the following combinations: *A*. *fumigatus* vs. *C*. *cladosporioides*, *A*. *fumigatus* vs. *A*. *alternata* and *C*. *cladosporoides* vs. *A*. *alternata*. Paired t-test performed between *A*. *fumigatus* and *A*. *alternata* had the null and alternate hypothesis as H_0_: µ_*AF*_ − µ_*AA*_ = 0, H_a_: µ_*AF*_ − µ_*AA*_ > 0 at a significance level of 0.05. Similarly, paired t-test was performed for the other two combinations as well. Additionally, the One-way ANOVA followed by Games-Howell post hoc test was performed using SPSS (IBM SPSS version 21) to further determine and prove the difference in the concentration distribution among the three allergenic fungi by analyzing their variance.

In order to determine the influence of the meteorological parameters such as temperature (°C), relative humidity (%), wind speed (kmh^−1^), wind gust (kmh^−1^) and precipitation (mm) on the occurrence of the analyzed allergenic fungi, a correlation matrix analysis based on linear regression (p < 0.05) was performed. The meteorological data (temperature, relative humidity, wind speed and precipitation) was obtained from the air quality database of the central pollution control board of India (CPCB).

### Morphological characterization of allergenic fungal spores

The morphological characteristics of the allergenic fungal spores were investigated using scanning electron microscopy (SEM) by following the protocol reported by Priyamvada *et al*.^74^. The fungi were grown from the PM_10_ collected on the filter paper over a year (June 2014 – July 2015) using the Sabouraud dextrose broth. Fungal spores were then extracted from the conidia under sterile conditions and then subjected to SEM imaging. The detailed procedure of spore extraction from the fungi is available in the supplementary section (see supplementary method and Fig. S4). The SEM analysis was performed for both the spore-bearing conidiophore and spore-released conidiophore. The glass slide containing the fungal spores was sputtered with a thin layer of gold to make the sample surface conducive for obtaining the secondary electron images of the spores under investigation^74, 75^. SEM images were obtained from the sophisticated analytical instrumentation facility (SAIF), IIT Madras using the Quanta FEG 200, FEI SEM instrument. The fungi grown from each of the filter paper were then subjected to DNA analysis to identify the individual fungal type.

### Allergy epidemiology study design

The cross-sectional epidemiological questionnaire survey was performed to assess the prevalence of allergic rhinitis to the inhabitants, belonging to a residential campus. And thus with this information, the association between PBAPs and allergies were established. The study was conducted amongst the residents visiting the hospital for general consultation and treatment during Aug 2013 to July 2014. The survey was conducted on “anonymous and voluntary” basis and around 1220 patients took part in the survey. The structured questionnaire requested the following information from the patients: demographic data (age, gender, campus residence), clinical features of asthma and rhinitis (frequency and severity of the symptoms, time of occurrence and duration of the allergies), the predominant and all the common occurrence of the symptoms of allergy, information on the occurrence of seasonal allergies, information on the co-morbid allergies and also on the campus specificity of the allergies. Note that the data collected for allergy survey does not coincide with the period of bioaerosol sampling. However, considering cyclic and systematic nature of the synoptic scale weather pattern over this region these two dataset can be combined for the preliminary scientific conclusion regarding role of observed fungal spores in allergies.

### Data Availability

All data generated and analyzed in this study are included in this article (and its Supplementary Information files).

## Electronic supplementary material

Supplementary Information
