# Supplementary material for: Seasonal variation of the dominant allergenic fungal aerosols – One year study from southern Indian region
Source: Sci Rep. 2017 Sep 11;7:11171. doi: 10.1038/s41598-017-11727-7 (PMC5593913; doi:10.1038/s41598-017-11727-7)
Supplement: Supplementary file 1 — Supplementary Information [file 41598_2017_11727_MOESM1_ESM.pdf]

Supplementary information

Seasonal variation of the dominant allergenic fungal aerosols – One year study from southern Indian region

Hema Priyamvada, [1,*] Raj Kamal Singh, [1] Akila M., [1] Ravikrishna, R., [2] Rama Shanker Verma, [3] & Sachin S. Gunthe[1,*]

[1] EWRE Division, Department of Civil Engineering, Indian Institute of Technology Madras, Chennai – 36, India

[2] Department of Chemical Engineering, Indian Institute of Technology Madras, Chennai – 36, India

[3] Department of Biotechnology, Indian Institute of Technology Madras, Chennai – 36, India

* Correspondence should be addressed to: Hema Priyamvada (hema8689@gmail.com), Sachin S. Gunthe (s.gunthe@iitm.ac.in)

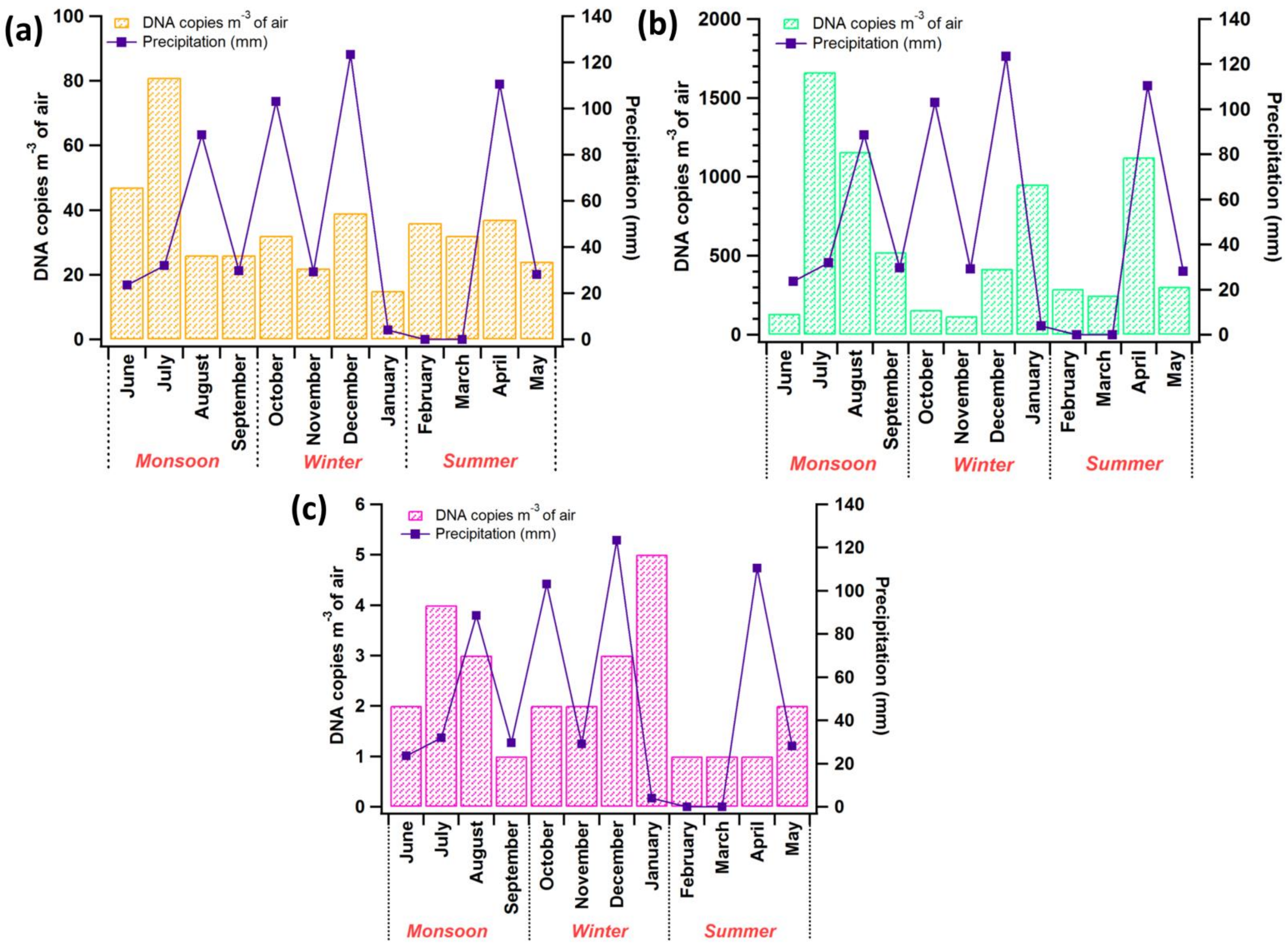

Figure S1. Relationship between precipitation (accumulated rainfall; mm) and allergenic fungi concentrations – *A. fumigatus* (a), *C. cladosporioides* (b), and *A. alternata* (c) (DNA copies m^{-3} of air). Precipitation was seen to influence only the concentration of *C. cladosporioides (c)*.

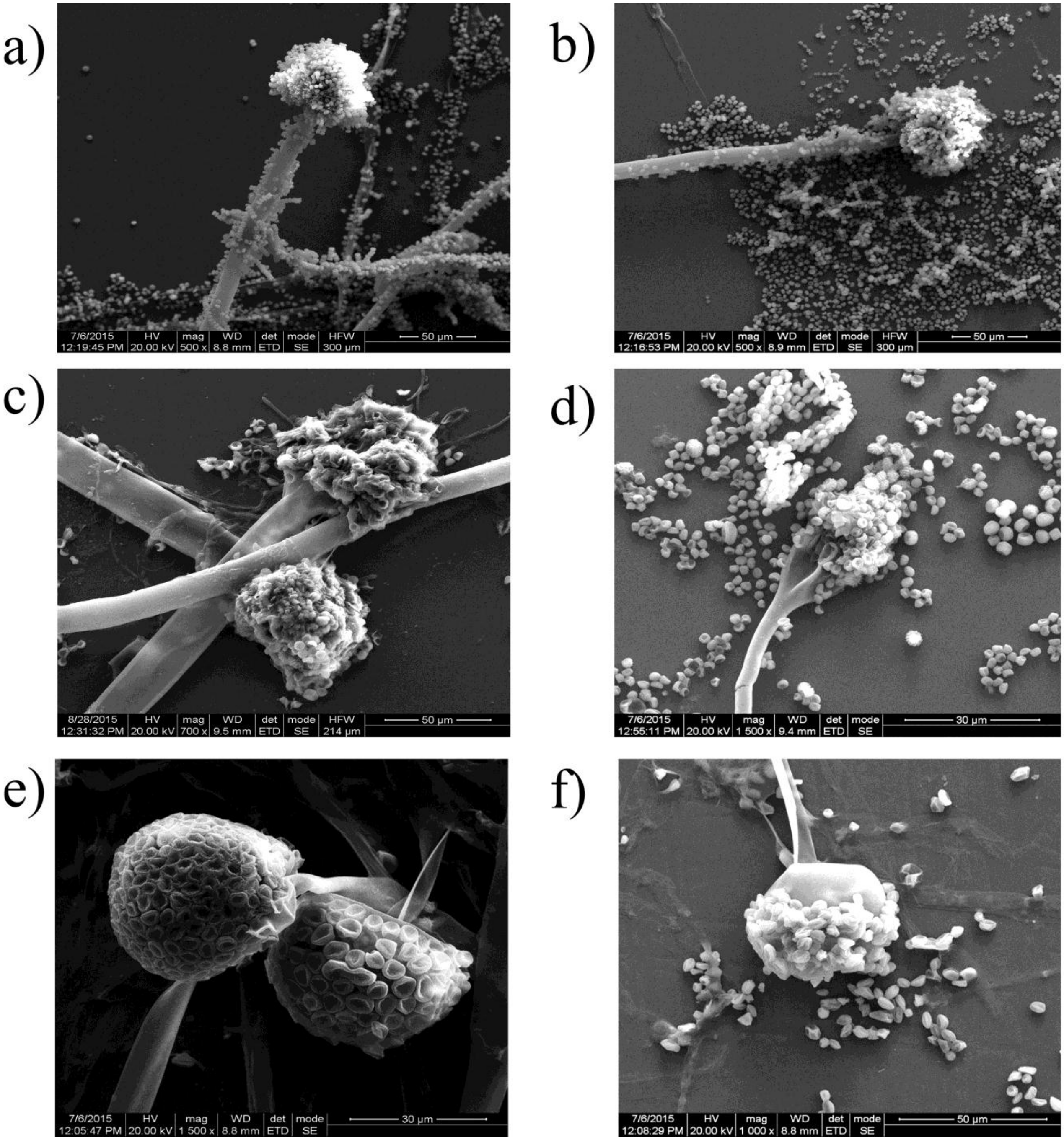
a)
7/6/2015 12:19:45 PM | HV 20.00 kV | mag 500 x | WD 8.8 mm | det ETD | mode SE | HFW 300 µm | 50 µm
b)
7/6/2015 12:16:53 PM | HV 20.00 kV | mag 500 x | WD 8.9 mm | det ETD | mode SE | HFW 300 µm | 50 µm
c)
8/28/2015 12:31:32 PM | HV 20.00 kV | mag 700 x | WD 9.5 mm | det ETD | mode SE | HFW 214 µm | 50 µm
d)
7/6/2015 12:55:11 PM | HV 20.00 kV | mag 1 500 x | WD 9.4 mm | det ETD | mode SE | 30 µm
e)
7/6/2015 12:05:47 PM | HV 20.00 kV | mag 1 500 x | WD 8.8 mm | det ETD | mode SE | 30 µm
f)
7/6/2015 12:08:29 PM | HV 20.00 kV | mag 1 000 x | WD 8.8 mm | det ETD | mode SE | 50 µm

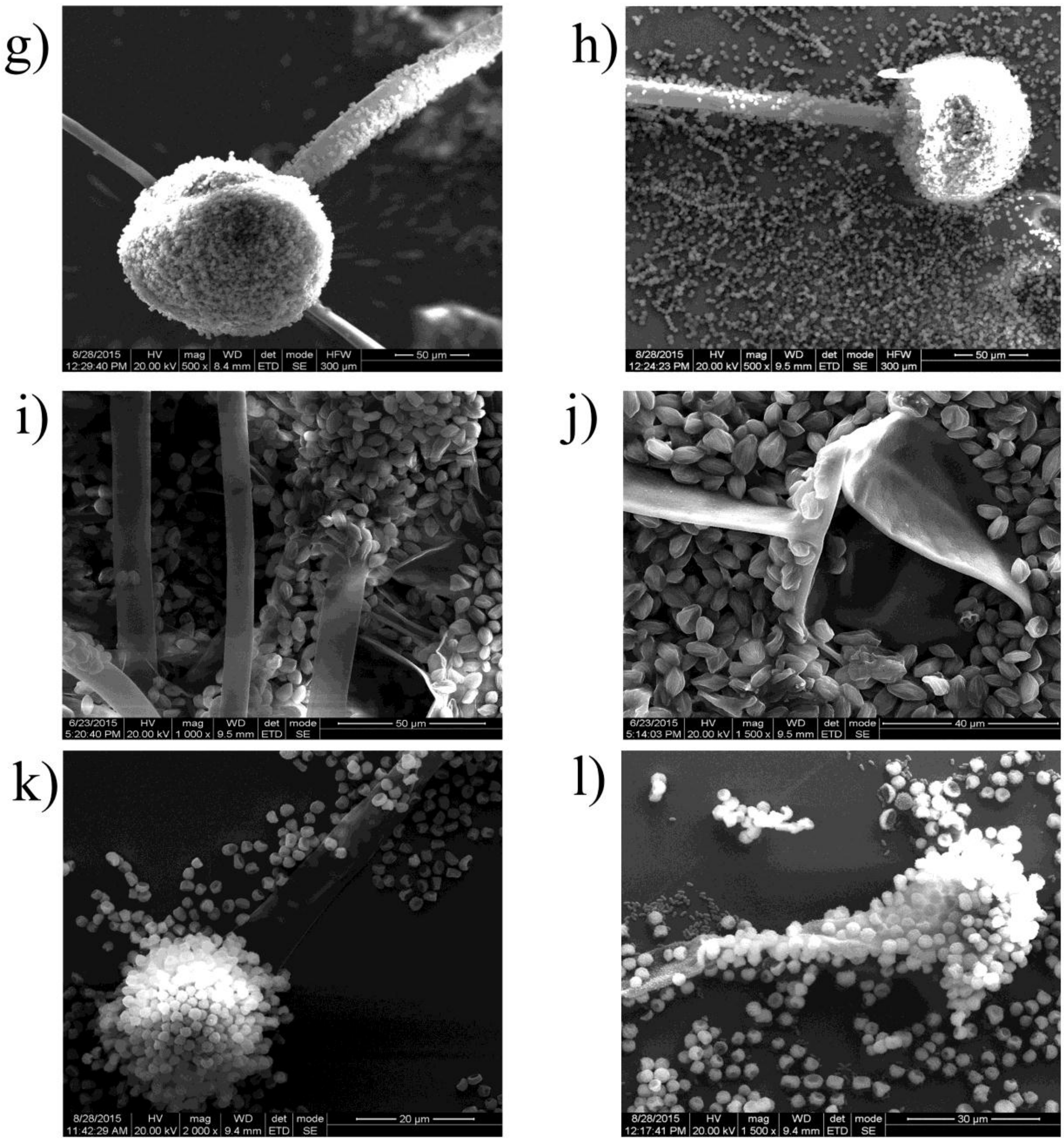

Figure S2. SEM images of conidia and conidiophore of allergenic fungi before and after the release of spores. *A. fumigatus* before (a) and after (b) spore release, *Rhizopus sp.* before (c) and after (d) spore release, *Aspergillus sp.* before (e) and after (f) spore release, *Epicoccum sp.* before (g) and after (h) spore release, *A. rhizopus* before (i) and after (j) spore release, *A. niger* before (k) and after (l) spore release.

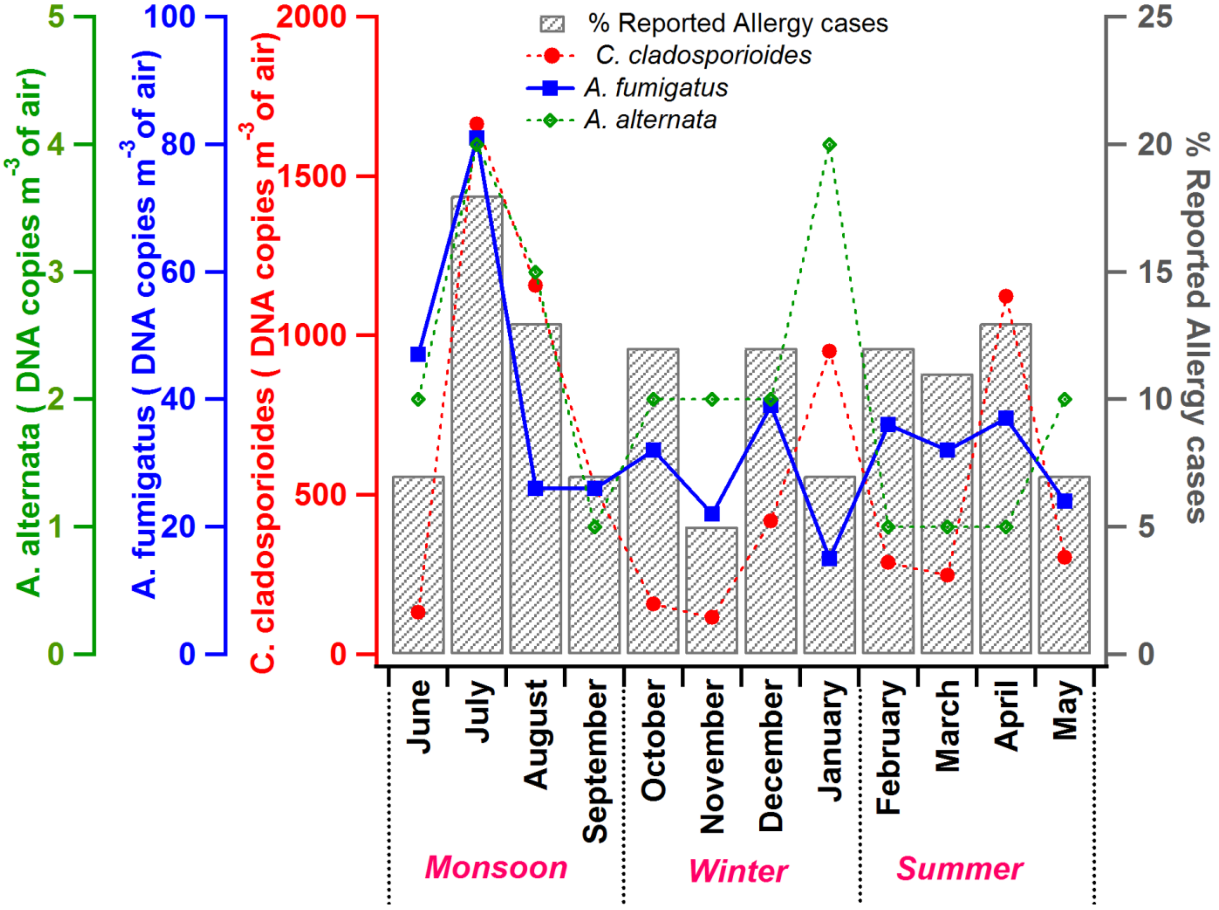

Figure S3. Relationship between the allergenic fungi (*A. fumigatus*, *C. cladosporioides,* and *A. alternata;* DNA copies m^{-3} of air) and the allergy cases reported (%) over the study region. Occurrence of allergy cases was seen to closely follow the trend of occurrence of *A. fumigatus.*

Methods

Morphological characterization of allergenic fungal spores. One quadrant (1cm×1cm) of the sampled air filter containing the PM_{10} was incubated in test tube containing the sabouraud dextrose broth (HiMedia, India) in a rotary incubator shaker (Orbitek ®, India) at 30˚C, 75 RPM from 48- 72 hrs. Visible growth of fungi was seen to occur after 24 hrs of incubation. Few exemplary images of the fungi grown on filter paper are provided below (Fig. S4). Subsequently, the condia and conidiophore were extracted at 48 and 72 hrs from all the incubated samples in order to perform the SEM analysis of the fungi under two conditions, (i) conidiophore before spore release and (ii) conidiophore after spore release. The fungal spores were then carefully extracted using a sterile scalpel and forceps. The extracted spores were carefully transferred to a glass slide containing a drop (2µl) of nuclease-free water. The slide was air dried before proceeding with the SEM analysis.

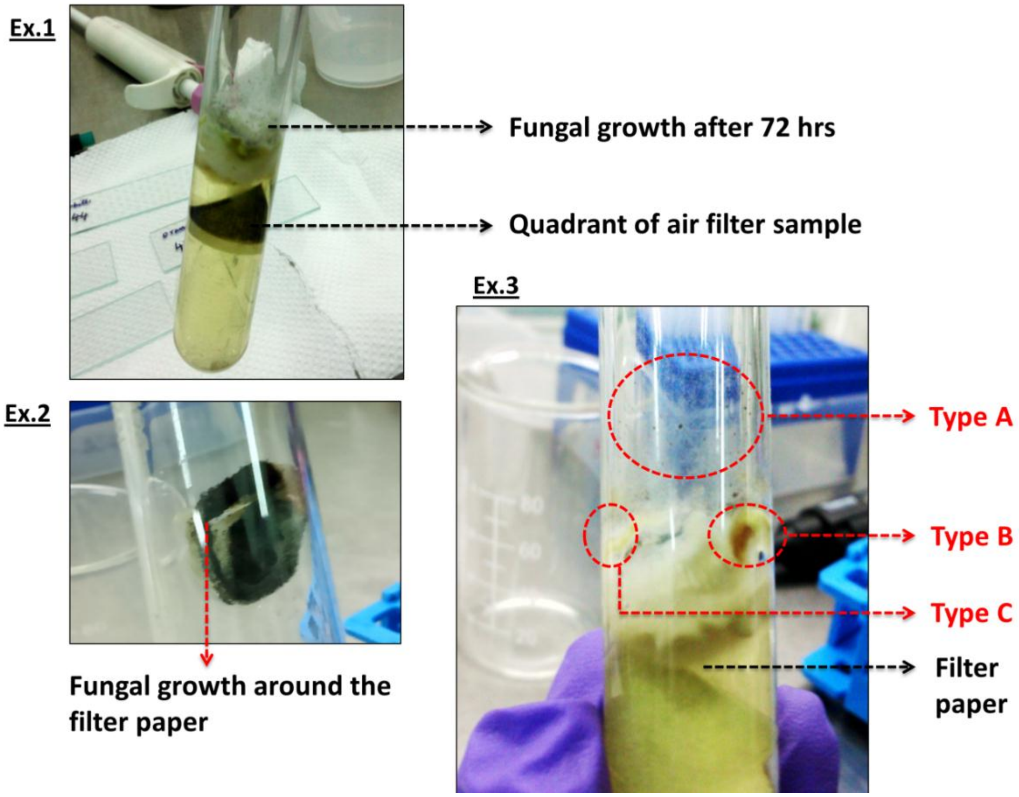

Figure S4. Exemplary images depicting the growth of fungi on the air filter samples incubated in the growth media. Here, three examples are shown as Ex. 1, 2, and 3. Ex.1 clearly shows the fungi grown from the filter sample. Ex.2 shows the 72hrs growth of fungi over the filter paper. Here we can see

the profuse growth of fungi surrounding the air filter sample. Ex.3 depicts three different types of fungi represented as types A, B, and C found growing from the filter sample.

Fungi			p values
Games-Howell	*AF*	*CC*	0.008
		AA	0.000
	CC	*AF*	0.008
		AA	0.005
	AA	*AF*	0.000
		CC	0.005

Table S1. The p-values obtained from One-way ANOVA-Games-Howell post hoc test. *AF - A. fumigatus, CC – C. cladosporioides, and AA - A. alternaria.* All of the three fungi were found to have significant difference in their concentration distribution.
